# Supplementary material for: Amorphous alloys surpass E/10 strength limit at extreme strain rates
Source: Nat Commun. 2024 Feb 26;15:1717. doi: 10.1038/s41467-024-45472-z (PMC10894860; doi:10.1038/s41467-024-45472-z)
Supplement: Supplementary file 1 — Supplementary Information [file 41467_2024_45472_MOESM1_ESM.pdf]

Supplementary Information for

Amorphous alloys surpass E/10 strength limit at extreme strain rates

Wenqing Zhu[1*], Zhi Li[2*], Hua Shu[3], Huajian Gao[2, 4†] and Xiaoding Wei[1, 5†]

[1] State Key Laboratory for Turbulence and Complex System, Department of Mechanics and Engineering Science, College of Engineering, Peking University, Beijing 100871, China

[2] Institute of High Performance Computing, Agency for Science, Technology and Research (A*STAR), Singapore, 138632, Republic of Singapore

[3] Shanghai Institute of Laser Plasma, China Academy of Engineering Physics, Shanghai, 201102, China

[4] School of Mechanical and Aerospace Engineering, College of Engineering, Nanyang Technological University, 70 Nanyang Drive, 637457, Singapore

[5] Peking University Nanchang Innovation Institute, Nanchang 330000, China

[*]These authors contributed equally: Wenqing Zhu, Zhi Li

[†]These authors jointly supervised this work: Huajian Gao, Xiaoding Wei

e-mail: huajian.gao@ntu.edu.sg (H.G.); xdwei@pku.edu.cn (X.W.)

I. Supplementary Figures and Tables

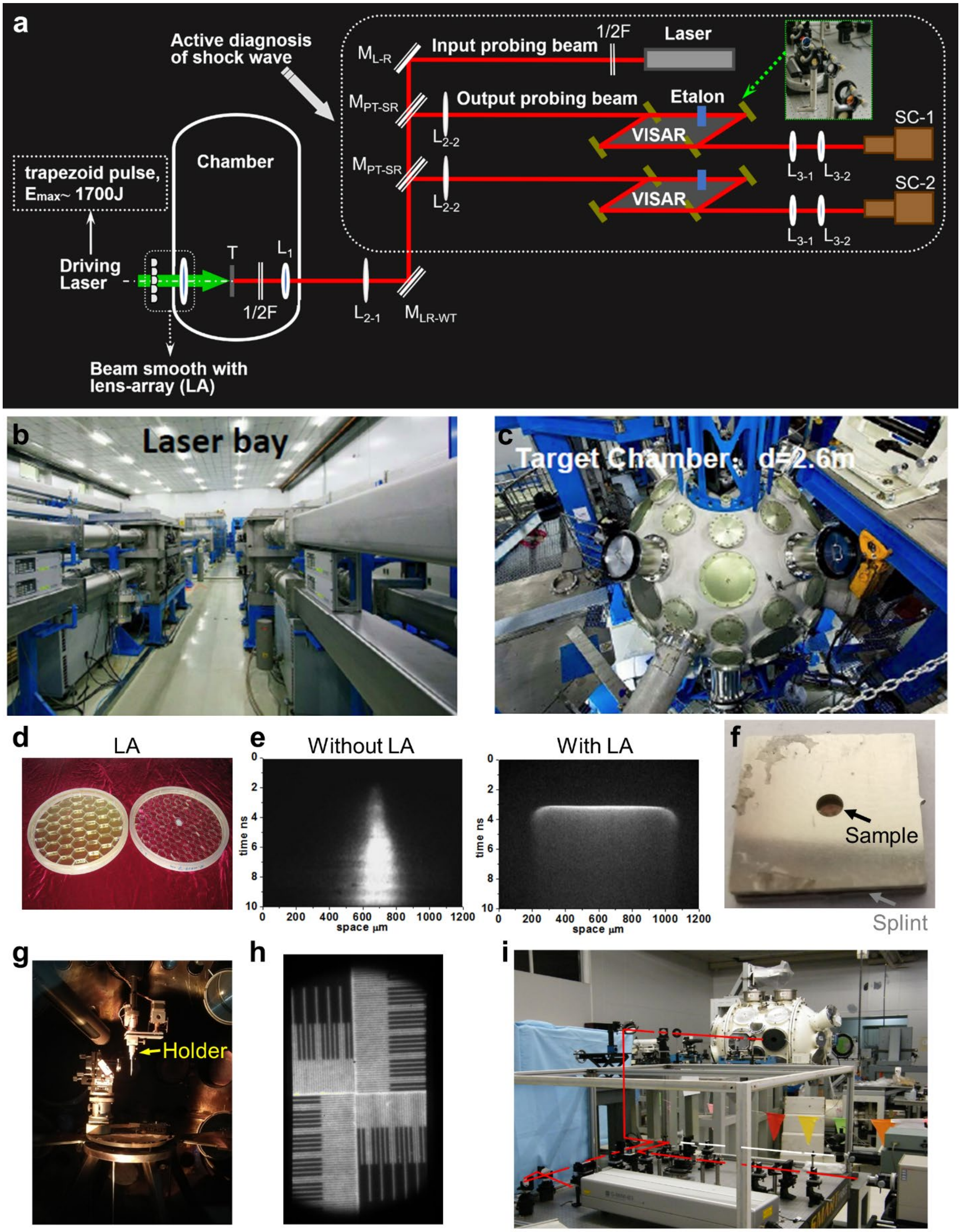

Supplementary Fig. 1. Details on the laser-induced shock experiments. a, Schematic of the experimental setup consisting of the laser source, the sample stage, and the VISAR diagnosis system. The first part of the imaging system (L1) produces a magnified real image. L1 is composed of four achromatic lenses. This combination has a 152 mm focal length and F/3 focal ratio. The magnification of this stage is 14.55×. L2-1 recollimates the beam for the path segment leading from the intermediate image and through a periscope system onto the optical table. It reaches a specially designed beam splitter $M_{LR\text{-}WT}$, which is a dichroic mirror. The probe laser (660 nm) is reflected from $M_{LR\text{-}WT}$ and reaches L2-2. The M-Z interferometers are positioned with precision so that the output images, which are generated 1100 mm beyond lens L2-2, coincide simultaneously with the output beam splitter in the interferometer. L3-1 recollimates the beam for the path segment leading from the output images and reaches L3-2. The output image is formed in the slit of the streak camera (SC-1 and SC-2). Note that the VISAR interferes with the same signal at two different times. The total magnification of the imaging system is 11×. **b, c,** Pictures of the laser bay and target chamber, respectively. The lens array (LA) (**d**) is used to eliminate the large-scale spatial modulation and to obtain a flat-topped profile on the focal plane (**e**). Sample (**f**) is fixed by a splint at its hole center. The splint is then fixed by the holder (**g**) in the target chamber for each laser shock test. **h**, Resolution pattern image shows a spatial resolution of ~ 7 μm for the two-channel VISAR. **i**, Picture of the VISAR optical system. The time resolution of the VISAR is ~ 20 ps.

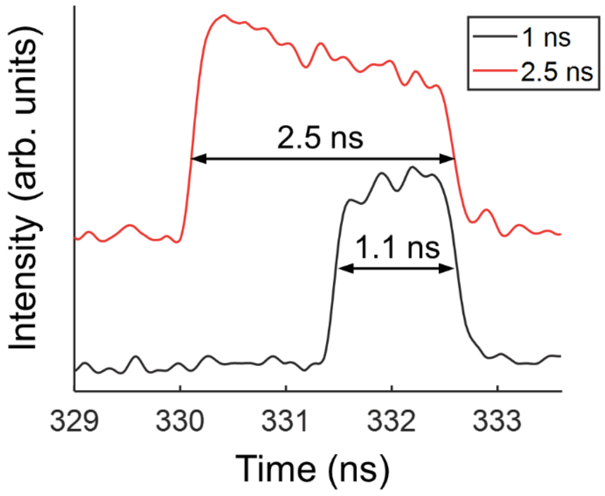

Supplementary Fig. 2. Representative input laser profiles with durations of 1 ns and 2.5 ns.

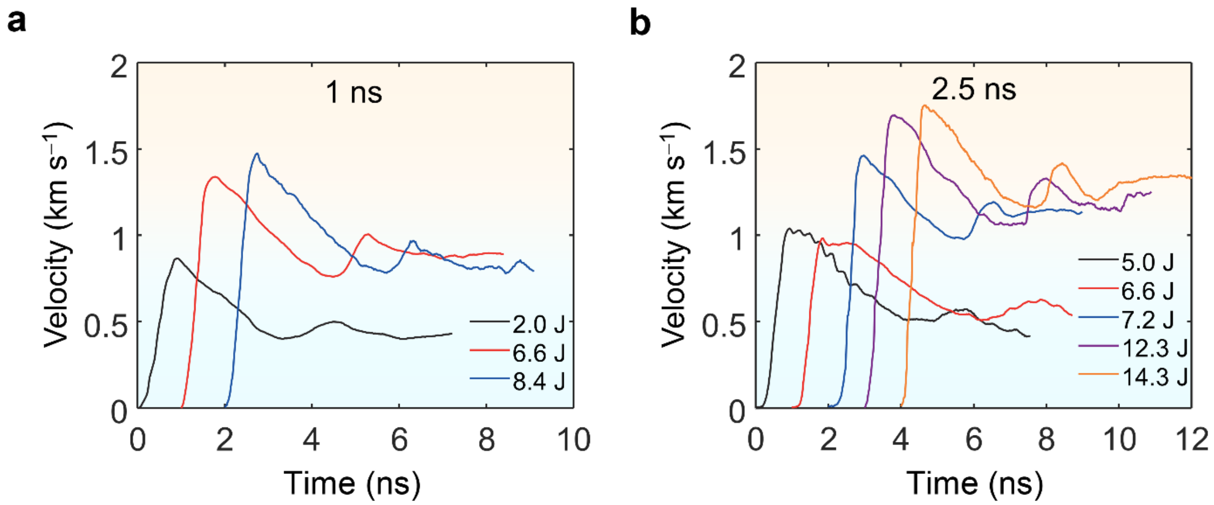

Supplementary Fig. 3. Histories of the free surface velocities for samples tested by laser pulses with durations of 1 ns (a) and 2.5 ns (b), respectively.

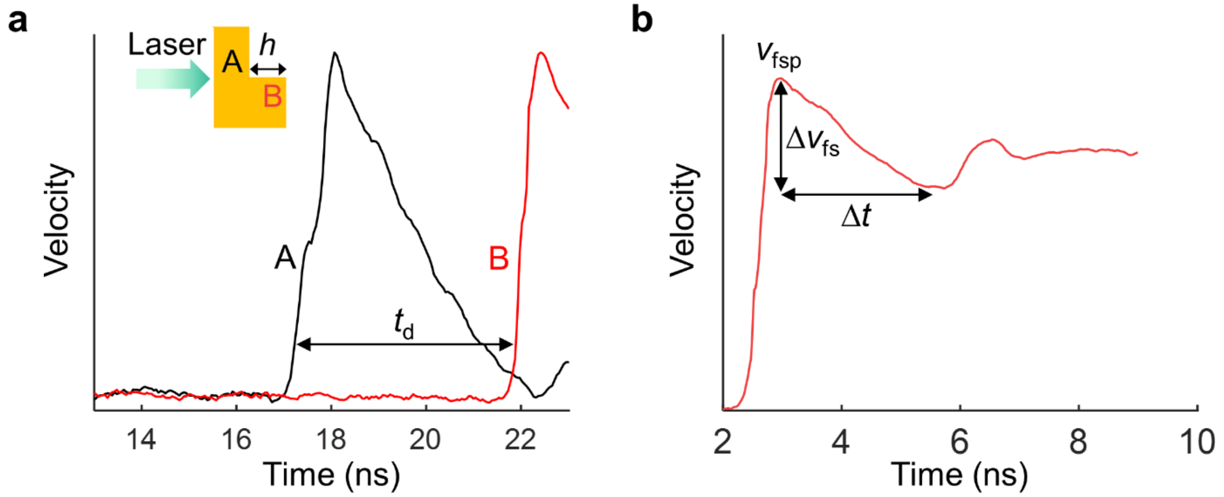

Supplementary Fig. 4. Calculation of the spall strength and strain rate. a, Schematic diagram of the measurement of the longitudinal speed of sound by shock tests on samples with a step on the rear surface. **b,** Typical FSV curve used to extract the spall strength and tensile strain rate.

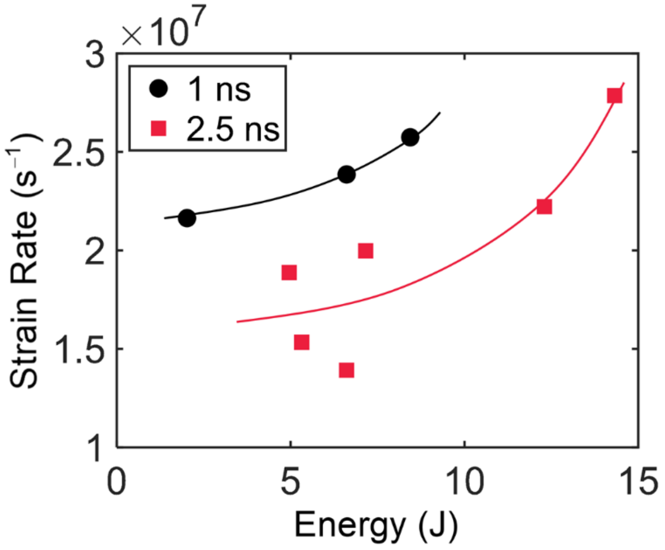

Supplementary Fig. 5. Tensile strain rate vs. input energy. The tensile strain rate increases with increasing laser power where either the energy rises or the duration becomes shorter.

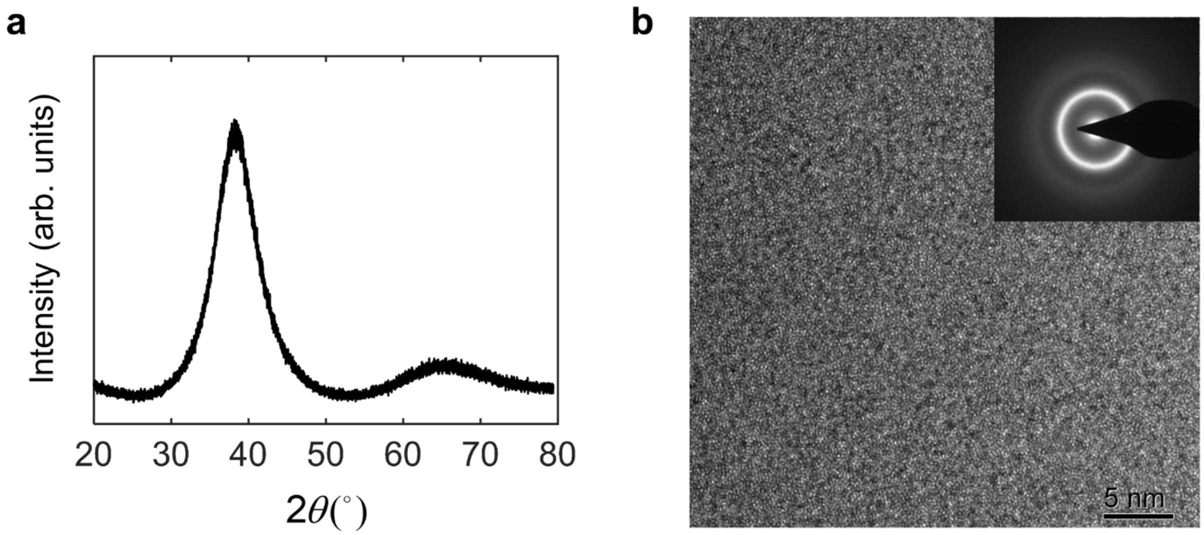

Supplementary Fig. 6. Structural characterizations of the as-cast MG samples confirm the glassy state. a, X-ray diffraction (XRD) spectrum. **b,** High-resolution transmission electron microscope (HRTEM) image and the selected-area electron diffraction (SAED) pattern (inset).

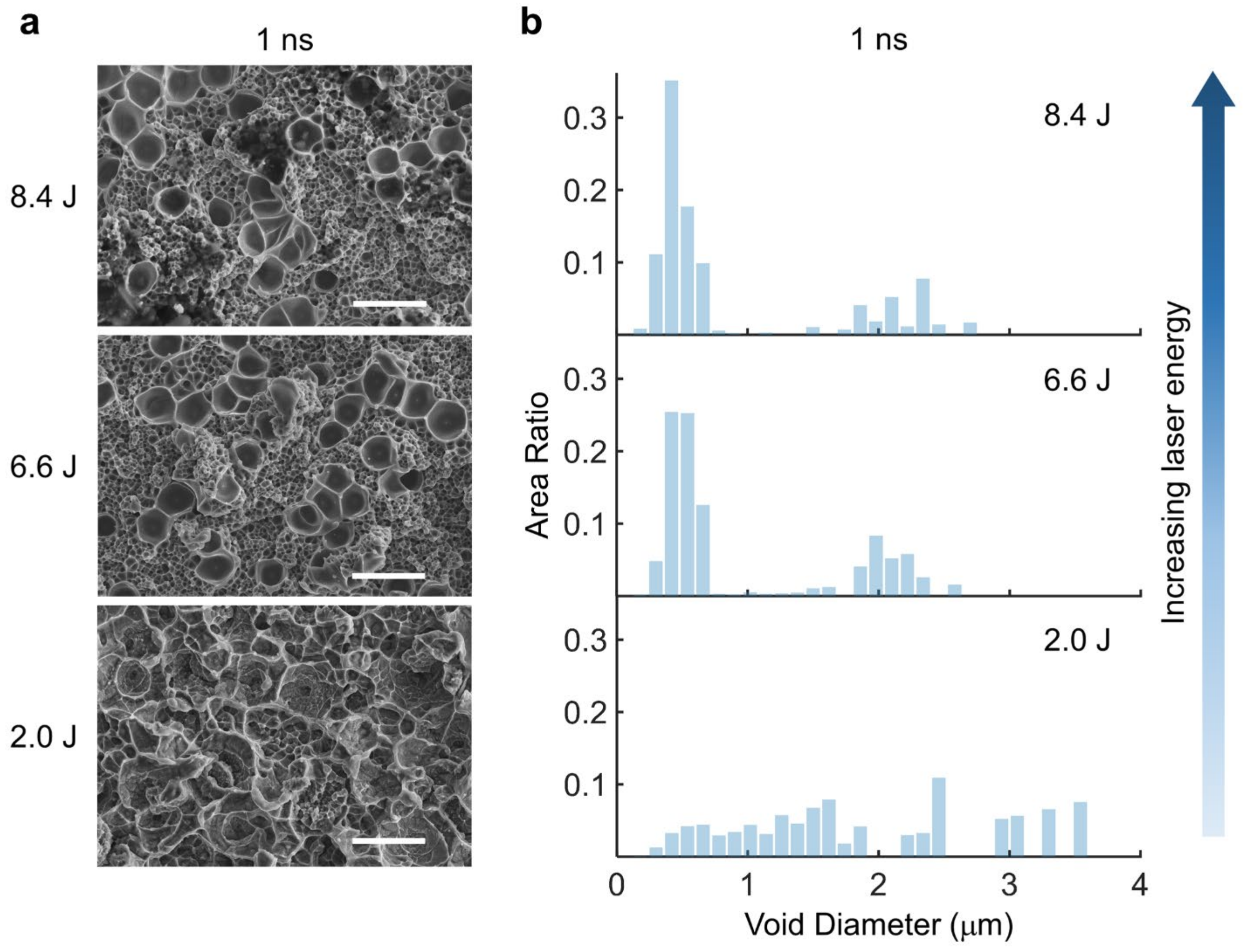

Supplementary Fig. 7. SEM micrographs and statistics of the void distribution on the spall surface dependent on the laser energy inputs. a, SEM micrographs of the spall surfaces of the samples tested by the laser with 1 ns duration but different input energies (from bottom to top: 2.0 J, 6.6 J, and 8.4 J). Scale bar, 5 μm. **b**, Histograms show the statistics of the void diameters in the micrographs in **a**.

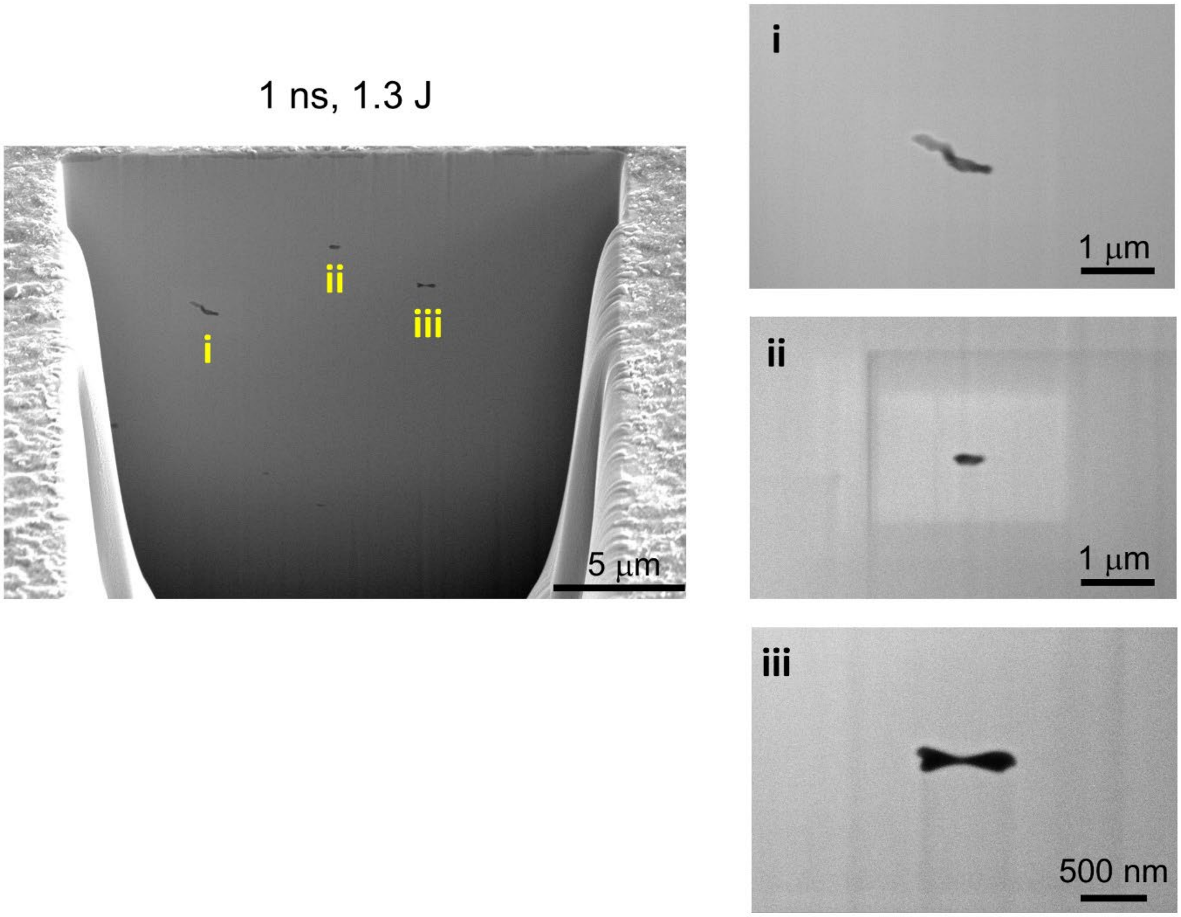

Supplementary Fig. 8. SEM micrographs show nanovoids underneath the back surface of the sample (tested by the laser with 1 ns pulse duration and 1.3 J energy, but did not spall).

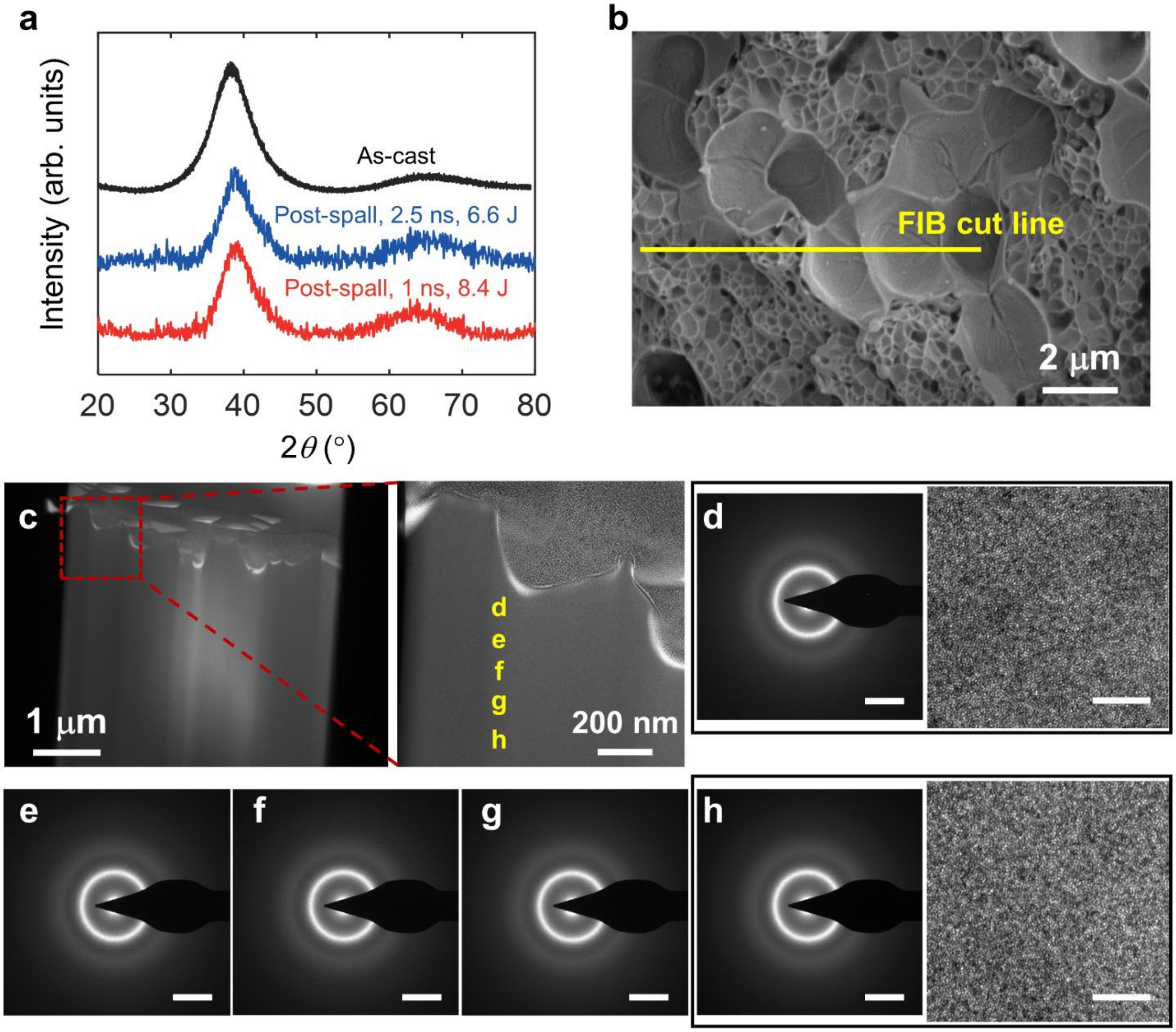

Supplementary Fig. 9. Structural characterizations of the post-spall MG samples. a, XRD spectra of one as-casted sample and two post-spall samples, showing similar typical broadened diffuse humps before and after tests. **b,** SEM image shows the location where the focus ion beam was used to cut into the spalled surface (of the sample tested by the laser with 1 ns duration and 8.4 J energy) to perform TEM characterization. of the spall plane showing the cross section for TEM prepared by the focused ion beam. **c,** Low-magnification TEM image shows the locations (labeled d-h**)** where selected-area electron diffraction (SAED) was performed. **d-h,** SAED halo patterns at different locations under the spall plane. HRTEM images for locations **d** and **h** are included to highlight the amorphous state. Scale bars for SAED and HRTEM images are 5 1/nm and 5 nm, respectively.

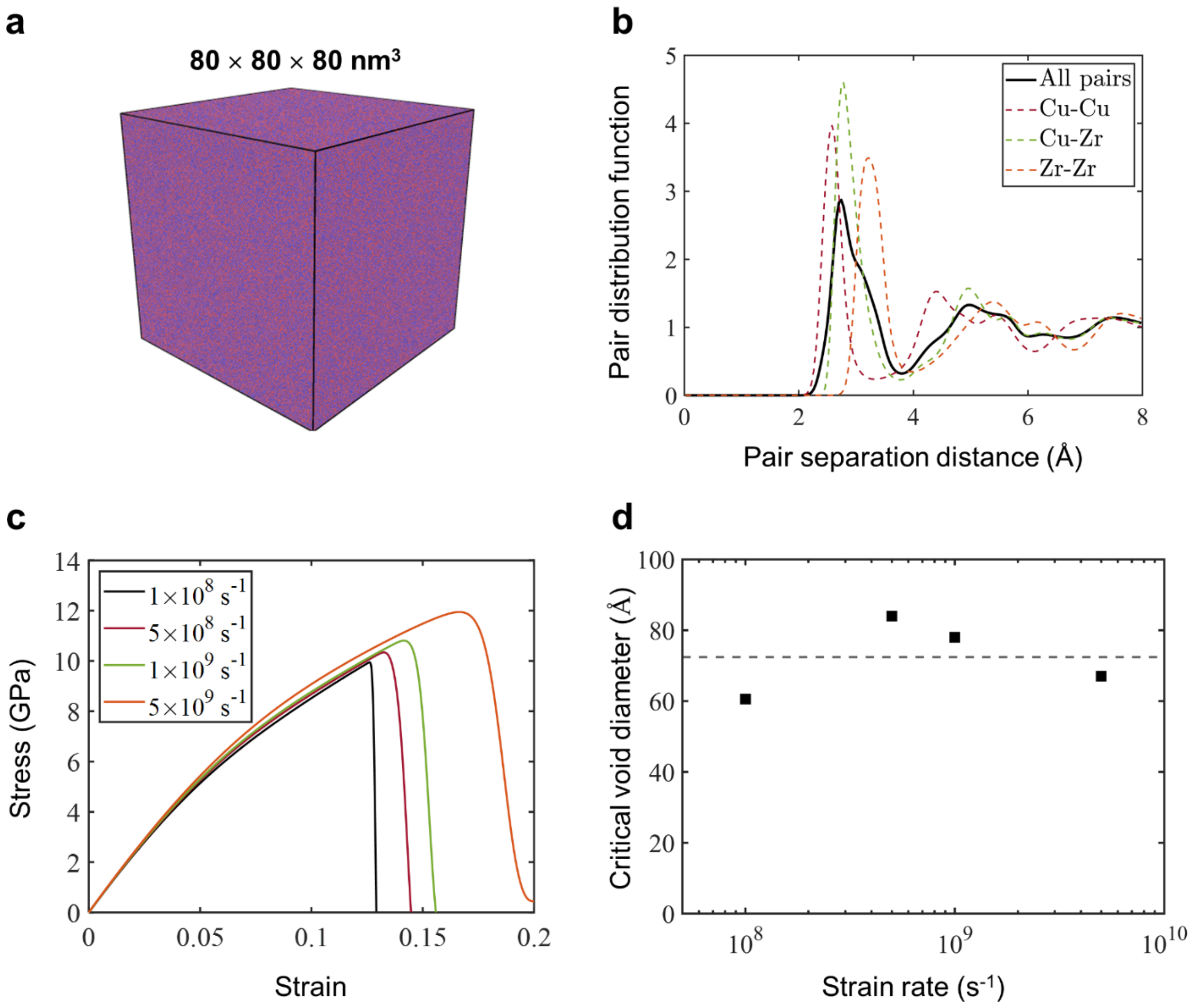

Supplementary Fig. 10. Large scale molecular dynamics simulations of the uniaxial strain tensile tests on $Cu_{50}Zr_{50}$. a, The Cu_{50} Zr_{50} model with a dimension of 80×80×80 nm^3 used for the simulations. Red atoms represent Cu and purple atoms represent Zr. **b**, The radial pair distribution function for the CuZr MG model. **c**, Stress-strain curves for Cu_{50} Zr_{50} MG model at strain rates ranging from 1×10^8 s^{-1} to 5×10^9 s^{-1} under uniaxial tensile strain. **d**, When the model is at its peak stress, the maximum void diameters are approximately 7.3 nm at different strain rates.

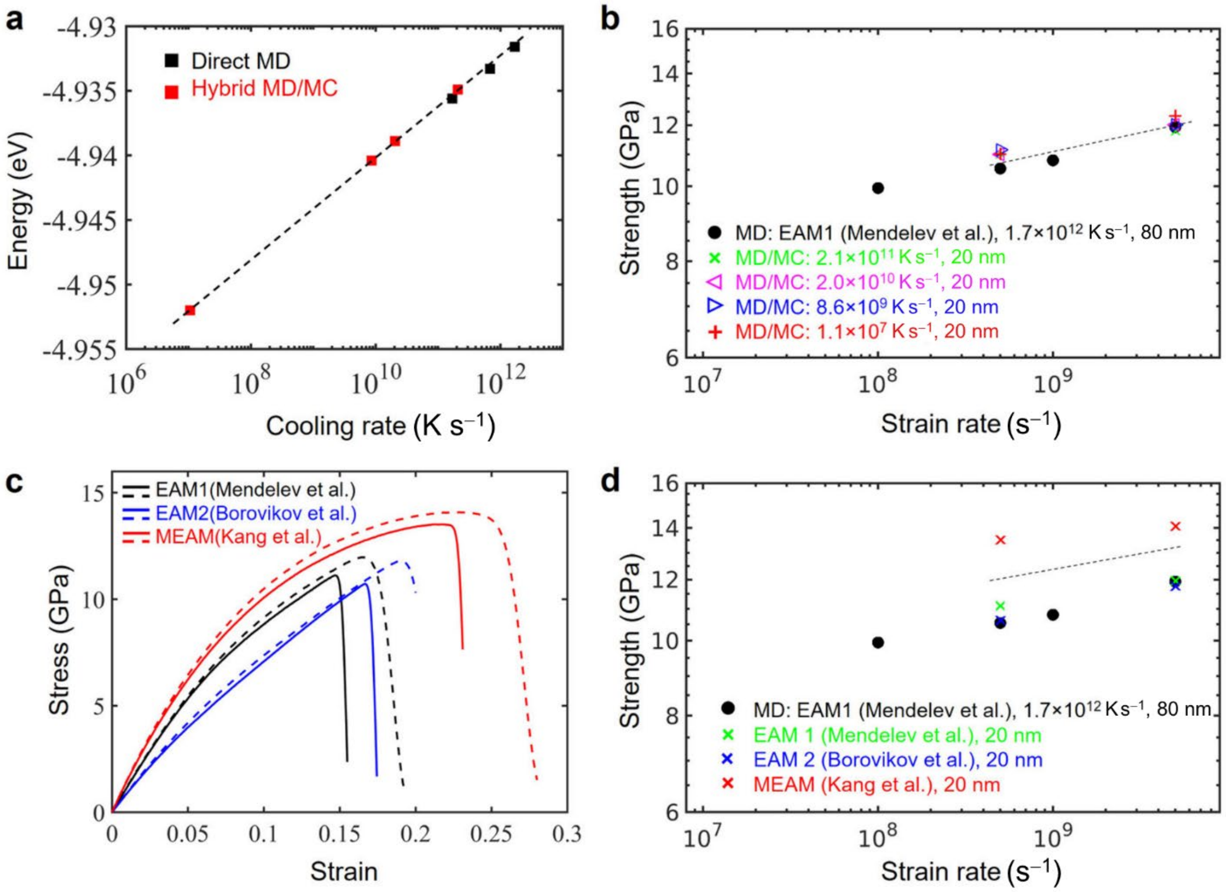

Supplementary Fig. 11. Effects of interatomic potentials and cooling rates on MD simulations. a, Calculated energy per atom at 300 K vs. effective cooling rate for the Cu-Zr models (20×20×20 nm^3 in size, containing ~0.5 million atoms). Red dots represent results for the much lower effective cooling rates (2.1×10^{11} K s^{-1}, 2.0×10^{10} K s^{-1}, 8.6×10^9 K s^{-1} and 1.1×10^7 K s^{-1}). **b,** Data of tensile strength vs. strain rate on the Cu-Zr models prepared at different cooling rates. The results show that the strain rate sensitivity is not sensitive to the cooling rate (black dots are the same results as in Supplementary Fig. 11). **c,** Stress-strain curves of the Cu-Zr model (20×20×20 nm^3, 1.7×10^{12} K s^{-1}) using different interatomic potentials at the strain rates of 5.0×10^8 s^{-1} (solid lines) and 5.0×10^9 s^{-1} (dashed lines). **d,** Data of tensile strength vs. tensile strain rate using different potential functions. The results suggest that the strain rate sensitivity is not sensitive to the choice of the interatomic potentials.

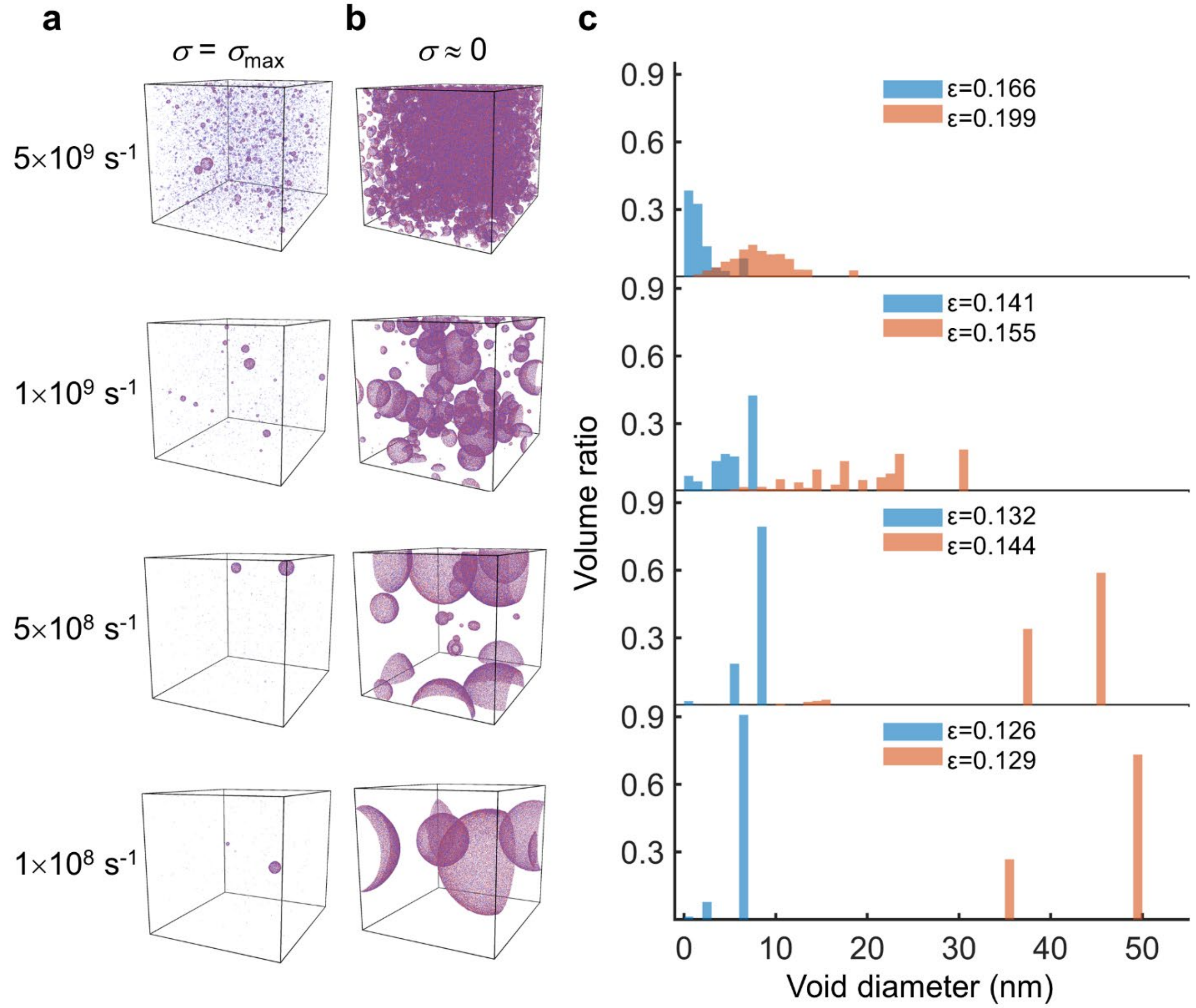

Supplementary Fig. 12. Rate-dependent void nucleation and growth revealed by molecular dynamics simulations. a, b, Void morphologies in $Cu_{50}Zr_{50}$ at the peak stress, and after material failure (i.e., when the stress decreases to zero), at different strain rates. **c**, Statistics of the void sizes at the peak stress and after material failure at different strain rates. The strains at two typical moments for each simulation are listed.

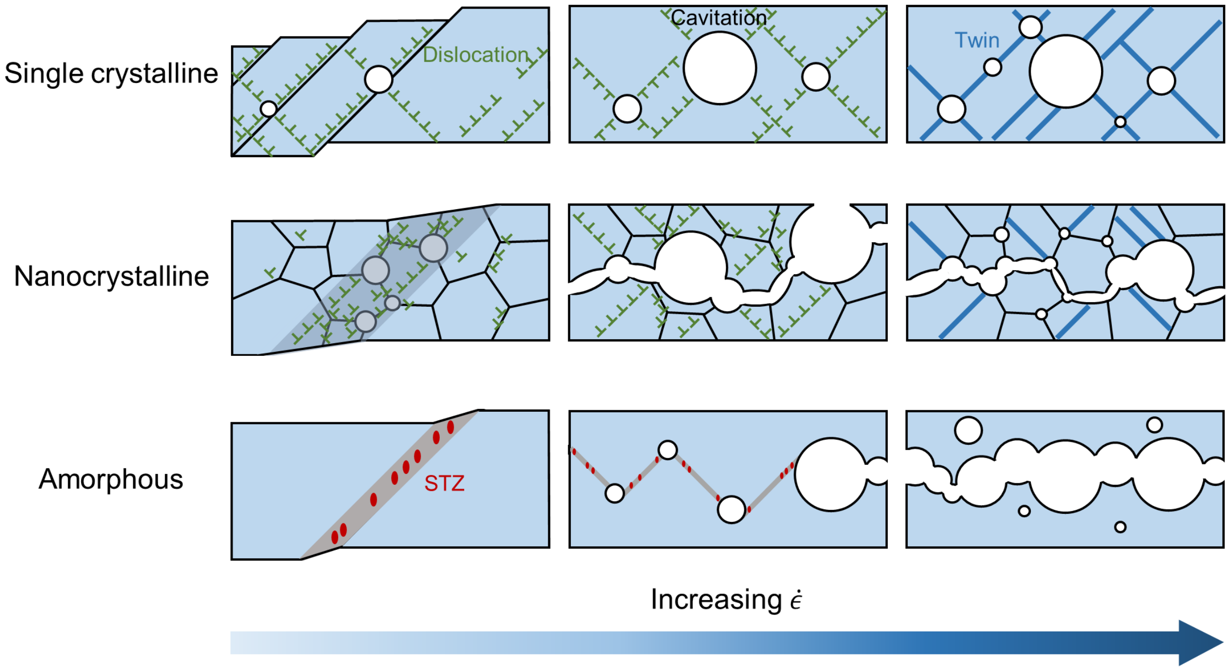

Supplementary Fig. 13. Failure mechanisms of crystalline and amorphous metals. Schematic diagrams of the representative failure mechanisms of metallic materials at various strain rates; arrows denote the tensile direction. Single crystals mainly fail due to slip bands mediated by multiple dislocations at quasi-static or low strain rates, cavitation induced by dislocations and emissions at high strain rates[1], and cavitation induced by intersections of twins at ultrahigh strain rates[2]. Nanocrystalline metals fail due to similar microscopic mechanisms, except that the grain boundaries and triple junctions act as sinks and sources for dislocation activities[2, 3]. In contrast, the main failure mechanism for amorphous metals is formation of shear bands at quasi-static or low strain rates[4]. At high strain rates, amorphous metals fail as the result of concurrent shear band and cavitation mechanisms[5]. Our study shows that amorphous metals fail mainly due to void growth and coalescence at ultrahigh strain rates (> 10^7 s^{-1}).

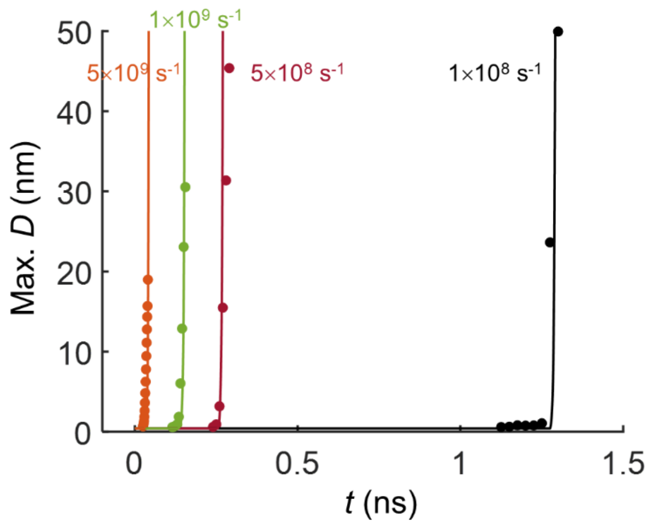

Supplementary Fig. 14. The temporal evolution of the maximum void diameter (symbols) at different strain rates during the MD simulation is in good agreement with the predictions of the kinetic model for void growth (lines).

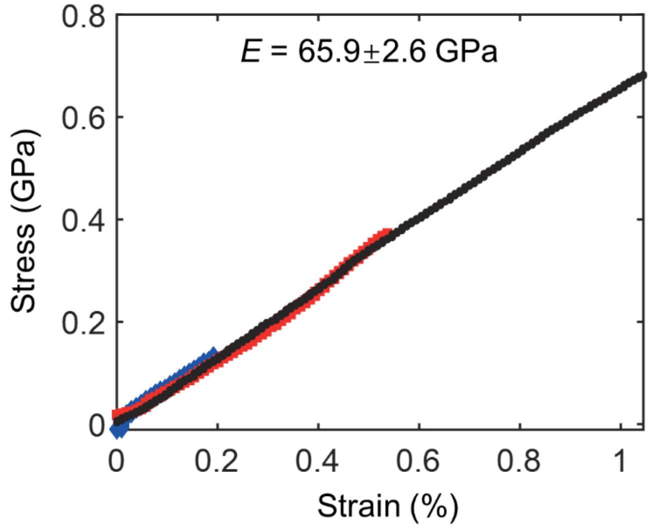

Supplementary Fig. 15. Stress-strain curves of the $Cu_{50}Zr_{50}$ MG ribbon under quasi-static uniaxial tensile tests.

Supplementary Table 1. Summary of results from previous studies and laser-induced shock tests in the present study.

ρ (kg m^{-3})	c (m s^{-1})	v_{fsp} (m s^{-1})	Δv_{fs} (m s^{-1})	Δt (ns)	σ_p (GPa)	σ_s* (GPa)	σ_s† (Gpa)	$\dot{\varepsilon}$ (s^{-1})	Refs.
6000	5184	347	254	279	5.4	4.0	/	8.8×10^4	Zhuang *et al.*
6762	4768	/	162	77	3.6	2.6	/	2.2×10^5	Escobedo *et al.*
		/	187	52	5.1	3.0	/	3.8×10^5	
		/	195	55	5.2	3.1	/	3.7×10^5	
		/	152	39	6.0	2.5	/	4.1×10^5	
6760	4750	360	206	224,	5.8	3.3	/	9.7×10^4	Ding *et al.*
		480	260	159,	7.7	4.2	/	1.7×10^5	
		520	310	175	8.4	5.0	/	1.9×10^5	
6940	4740	453	/	/	7.4	4.1	/	7.0×10^5	Tang *et al.*
		363	/	/	6.0	3.2	/	3.5×10^5	
7427	4487	867	466	2.4	14.4	7.8	8.2	2.2×10^7	Present study
		1340	578	2.7	22.3	9.6	10.0	2.4×10^7	
		992	399	2.9	16.5	6.6	6.9	1.5×10^7	
		1040	525	3.1	17.3	8.7	9.0	1.9×10^7	
		982	437	3.5	16.4	7.3	7.4	1.4×10^7	
		1695	638	3.2	28.2	10.6	10.9	2.2×10^7	
		1462	484	2.7	24.4	8.1	8.4	2.0×10^7	
		1753	525	2.1	29.2	8.7	9.3	2.8×10^7	
		1475	693	3.0	24.6	11.5	11.9	2.6×10^7	

*Evaluated based on the linear acoustic approximation $\sigma_s = \rho c \Delta v_{fs}/2$.

†Evaluated by the thickness correction formula $\sigma_s = \frac{\rho c}{(1 + c/c_B)}(\Delta v_{fs} + \delta)$; c_B is the bulk speed of sound, $\delta = |\dot{v}_1| h_{sp}\left(\frac{1}{c_B} - \frac{1}{c}\right)$ is the correction factor, $\dot{v}_1$ is the free surface velocity derivative ahead of the spall pulse, and $h_{sp} \approx 10$ μm is the thickness of the spalling layer (refs. 6, 7).

II. Supplementary Notes

(1) Derivations of the analytical solution of the kinetic model of void growth

Under the control of surface energy, the evolution of the void diameter $D(t)$ based on Eq. (1) can be reorganized as follows:

$$\dot{D} = \begin{cases} \frac{1+\nu}{1-\nu}\frac{mM\dot{\varepsilon}t}{3}D - 4m\gamma, t > t_0 \\ 0, t \le t_0 \end{cases}, \tag{1}$$

where $t_0 = \frac{12\gamma(1-\nu)}{D_0 M\dot{\varepsilon}(1+\nu)}$ is the time when the void starts to grow, given by the condition $\sigma_h = 4\gamma/D_0$; D_0 is the initial void diameter. The following form can be used for the solution to the above equation:

$$D(t) = \begin{cases} f(t)\exp\left[\frac{m(1+\nu)}{6(1-\nu)}M\dot{\varepsilon}\left(t^2 - t_0^2\right)\right], \ t > t_0 \\ D_0, \ t \le t_0 \end{cases}. \tag{2}$$

Thus, for $t \ge t_0$, we have

$$\dot{D} = \frac{m(1+\nu)}{3(1-\nu)}M\dot{\varepsilon}tD + f'(t)\exp\left[\frac{m(1+\nu)}{6(1-\nu)}M\dot{\varepsilon}\left(t^2 - t_0^2\right)\right] = \frac{m(1+\nu)}{3(1-\nu)}M\dot{\varepsilon}tD - 4m\gamma, \tag{3}$$

which leads to the analytical expression for $f(t)$:

$$\begin{aligned} f(t) &= D_0 - 4m\gamma\exp\left[\frac{m(1+\nu)}{6(1-\nu)}M\dot{\varepsilon}t_0^2\right]\int_{t_0}^{t}\exp\left[-\frac{m(1+\nu)}{6(1-\nu)}M\dot{\varepsilon}x^2\right]dx \\ &= D_0 - 2\gamma\sqrt{\frac{6\pi m(1-\nu)}{M\dot{\varepsilon}(1+\nu)}}\exp\left[\frac{m(1+\nu)}{6(1-\nu)}M\dot{\varepsilon}t_0^2\right]\times \\ &\left[\operatorname{erf}\left(\sqrt{\frac{M\dot{\varepsilon}m(1+\nu)}{6(1-\nu)}}t\right) - \operatorname{erf}\left(\sqrt{\frac{M\dot{\varepsilon}m(1+\nu)}{6(1-\nu)}}t_0\right)\right] \end{aligned}. \tag{4}$$

Notably, the initial condition $D(t_0) = D_0$ has been applied above. Finally, we obtain the analytical solution for the void diameter evolution $D(t)$:

$$D(t) = \begin{cases} \left\{D_0\exp\left(-\frac{m}{6}\frac{1+\nu}{1-\nu}M\dot{\varepsilon}t_0^2\right) - 2\gamma\sqrt{\frac{6\pi m(1-\nu)}{M\dot{\varepsilon}(1+\nu)}}\left[\operatorname{erf}\left(\sqrt{\frac{M\dot{\varepsilon}m(1+\nu)}{6(1-\nu)}}t\right)\right.\right. \\ \left.\left. -\operatorname{erf}\left(\sqrt{\frac{M\dot{\varepsilon}m(1+\nu)}{6(1-\nu)}}t_0\right)\right]\right\}\exp\left(\frac{m}{6}\frac{1+\nu}{1-\nu}M\dot{\varepsilon}t^2\right), \ t > t_0 \\ D_0, \ t \le t_0 \end{cases} \tag{5}$$

(2) Critical void size from the mechanical instability analysis

The total energy of the system with a void of diameter D is $F = \pi D^2\gamma - \pi D^3\psi/6$, in which the first term is the surface energy, and the second term is the elastic strain energy, where ψ is the elastic strain energy density. Thus, the mechanical stability of the system breaks down when $\mathrm{d}F/\mathrm{d}D = 0$, which yields:

$$2D\gamma - \frac{D^2\psi}{2} = 0. \quad (6)$$

Therefore, the critical void diameter at the instability is $D_\mathrm{c} = 4\gamma/\psi$. The elastic strain energy density is approximately $\psi = M\varepsilon^2/2$ where M = 126.9 GPa is the P-wave modulus and $\varepsilon \sim 0.1$. Taking γ = 1.28 J/m^2 obtained from MD simulations, we estimate the critical void diameter $D_\mathrm{c} \approx$ 8.1 nm, which agrees remarkably well with the value of $D_\mathrm{c} \approx$ 7.3 nm obtained from the MD simulations.

Supplementary References

1. Bringa EM, Traiviratana S, Meyers MA. Void initiation in fcc metals: Effect of loading orientation and nanocrystalline effects. *Acta Materialia* **58**, 4458-4477 (2010).

2. Righi G*, et al.* Towards the ultimate strength of iron: spalling through laser shock. *Acta Materialia*, (2021).

3. Meyers MA, Mishra A, Benson DJ. Mechanical properties of nanocrystalline materials. *Progress in Materials Science* **51**, 427-556 (2006).

4. Greer AL, Cheng YQ, Ma E. Shear bands in metallic glasses. *Materials Science and Engineering: R: Reports* **74**, 71-132 (2013).

5. Tang XC*, et al.* Cup-cone structure in spallation of bulk metallic glasses. *Acta Materialia* **178**, 219-227 (2019).

6. Romanchenko VI, Stepanov GV. Dependence of the critical stresses on the loading time parameters during spall in copper, aluminum, and steel. *Journal of Applied Mechanics and Technical Physics* **21**, 555-561 (1980).

7. Kanel G. Spall fracture: methodological aspects, mechanisms and governing factors. *International Journal of Fracture* **163**, 173-191 (2010).
