# Supplementary material for: Amorphous alloys surpass E/10 strength limit at extreme strain rates
Source: Nat Commun. 2024 Feb 26;15:1717. doi: 10.1038/s41467-024-45472-z (PMC10894860; doi:10.1038/s41467-024-45472-z)
Supplement: Supplementary file 3 — Inventory of Supplementary Information [file 41467_2024_45472_MOESM3_ESM.pdf]

Inventory of Supplementary Information for

Amorphous alloys surpass E/10 strength limit at extreme strain rates

Wenqing Zhu[1*], Zhi Li[2*], Hua Shu[3], Huajian Gao[2, 4†] and Xiaoding Wei[1, 5†]

[1] State Key Laboratory for Turbulence and Complex System, Department of Mechanics and Engineering Science, College of Engineering, Peking University, Beijing 100871, China

[2] Institute of High Performance Computing, Agency for Science, Technology and Research (A*STAR), Singapore, 138632, Republic of Singapore

[3] Shanghai Institute of Laser Plasma, China Academy of Engineering Physics, Shanghai, 201102, China

[4] School of Mechanical and Aerospace Engineering, College of Engineering, Nanyang Technological University, 70 Nanyang Drive, 637457, Singapore

[5] Peking University Nanchang Innovation Institute, Nanchang 330000, China

[*]These authors contributed equally: Wenqing Zhu, Zhi Li

[†]These authors jointly supervised this work: Huajian Gao, Xiaoding Wei

e-mail: huajian.gao@ntu.edu.sg (H.G.); xdwei@pku.edu.cn (X.W.)

Supplementary Fig. 1 to 15

Supplementary Table 1

Supplementary Notes

Supplementary References
